# Cryo-EM structure of *Neurospora crassa* respiratory complex IV

**DOI:** 10.1107/S2052252519007486

**Published:** 2019-06-26

**Authors:** Thomas Bausewein, Stephan Nussberger, Werner Kühlbrandt

**Affiliations:** a Department of Structural Biology, Max-Planck-Institute of Biophysics, Max-von-Laue-Strasse 3, 60438 Frankfurt am Main, Germany; b Abteilung Biophysik, Institut für Biomaterialien und biomolekulare Systeme, Universität Stuttgart, Pfaffenwaldring 57, 70550 Stuttgart, Germany

**Keywords:** 3D reconstruction and image processing, automation, single-particle cryo-EM, structure determination, cryo-electron microscopy, cytochrome *c* oxidase, *Neurospora crassa*, respiratory complex IV

## Abstract

In fungi, the mitochondrial respiratory chain complexes (complexes I–IV) are responsible for oxidative phosphorylation, as in higher eukaryotes. Cryo-EM was used to identify a 200 kDa membrane protein from *Neurospora crassa* in lipid nanodiscs as cytochrome *c* oxidase (complex IV) and its structure was determined at 5.5 Å resolution. The map closely resembles the cryo-EM structure of complex IV from *Saccharomyces cerevisiae*. Its ten subunits are conserved in *S. cerevisiae* and *Bos taurus*, but other transmembrane subunits are missing. The different structure of the Cox5a subunit is typical for fungal complex IV and may affect the interaction with complex III in a respiratory supercomplex. Additional density was found between the matrix domains of the Cox4 and Cox5a subunits that appears to be specific to *N. crassa*.

## Abstract 2

The cryo-EM structure of *Neurospora crassa* respiratory complex IV was determined to 5.5 Å resolution and is compared with related structures from *Saccharomyces cerevisiae* and *Bos taurus*.

## 1. Introduction   

The mitochondrial respiratory chain consists of NADH dehydrogenase (complex I), succinate dehydrogenase (complex II), cytochrome *c* reductase (complex III) and cytochrome *c* oxidase (complex IV). Mitochondrial complex IV is a 200 kDa heme-copper oxidase with up to 14 different polypeptides (Balsa *et al.*, 2012 ▸). Complex IV catalyzes the reduction of molecular oxygen to water and conserves free energy by pumping protons across the mitochondrial inner membrane (Wikstrom, 1977 ▸). Owing to the high-resolution crystal structure of bovine heart complex IV (Tsukihara *et al.*, 1995 ▸, 1996 ▸), the molecular mechanisms of the three central subunits as a redox-coupled proton pump are well understood (Yoshikawa *et al.*, 2011 ▸; Shinzawa-Itoh *et al.*, 2007 ▸; Liang *et al.*, 2017 ▸). There is genetic evidence in yeast (Capaldi, 1990 ▸) that most of the peripheral subunits function as assembly factors of complex IV. Null mutants yield enzymes with reduced activities compared with the wild type.

In mammalian mitochondria, complexes I, III and IV are known to form supercomplexes of different compositions (Schäfer *et al.*, 2007 ▸; Gu *et al.*, 2016 ▸; Althoff *et al.*, 2011 ▸; Wu *et al.*, 2016 ▸; Sousa *et al.*, 2016 ▸; Letts *et al.*, 2016 ▸) that are sometimes referred to as respirasomes. Other eukaryotes and fungi have characteristic variants. These include the supercomplex of *Saccharomyces cerevisiae*, a symmetric assembly of complex III and complex IV that has recently been studied by high-resolution cryo-EM (Hartley *et al.*, 2019 ▸; Rathore *et al.*, 2019 ▸). In *S. cerevisiae* complex I is replaced by three non-proton-translocating alternative peripheral membrane NADH:ubiquinone oxidoreductases exposed on the inner or outer surface of the mitochondrial inner membrane (Joseph-Horne *et al.*, 2001 ▸; Iwata *et al.*, 2012 ▸; Feng *et al.*, 2012 ▸). Unlike *S. cerevisiae*, its close relative the filamentous fungus *Neurospora crassa* has a complex I (Videira, 1998 ▸; Marques *et al.*, 2005 ▸). Biochemical analysis of digitonin-solubilized mitochondria from *N. crassa* by blue native gel electrophoresis revealed assemblies of complexes I and IV comigrating with ATP synthase dimers, supercomplexes of complexes III and IV, and supercomplexes of complexes I, III and IV with different stoichiometries (Marques *et al.*, 2007 ▸). The low-resolution structures of *N. crassa* complex I and complex III have been studied by electron microscopy of negatively stained particles (Guénebaut *et al.*, 1997 ▸) or electron crystallography of two-dimensional crystals (Leonard *et al.*, 1987 ▸). However, the architecture of *N. crassa* complex IV or its respiratory supercomplexes have not been explored. We now report the 5.5 Å resolution structure of complex IV from *N. crassa* reconstituted into lipid nanodiscs, as determined by single-particle cryo-EM. Our results underline the conserved molecular mechanism by which complex IV facilitates the reduction of molecular oxygen to water in mammals and fungi.

## 2. Materials and methods   

### 2.1. Growth of cells and preparation of mitochondria   

*N. crassa* (strain GR-107) was grown and mitochondria were isolated as described previously (Sebald *et al.*, 1979 ▸; Künkele *et al.*, 1998 ▸; Ahting *et al.*, 1999 ▸). Briefly, 1 kg (wet weight) of hyphae were homogenized in 250 m*M* sucrose, 2 m*M* EDTA, 20 m*M* Tris pH 8.5, 1 m*M* PMSF in a Waring blender at 4°C. 1 kg of quartz sand was added and the cell walls were disrupted by passing the suspension twice through a corundum stone mill. Cellular residues were pelleted and discarded in two centrifugation steps (4000*g*) for 5 min at 4°C. The mitochondria were sedimented in 250 m*M* sucrose, 2 m*M* EDTA, 20 m*M* Tris pH 8.5, 1 m*M* PMSF at 17 000*g* for 80 min. This step was repeated to improve the purity. The isolated mitochondria were suspended in 250 m*M* sucrose, 20 m*M* Tris pH 8.5, 1 m*M* PMSF at a final protein concentration of 50 mg ml^−1^, shock-frozen in liquid nitrogen and stored at −20°C.

### 2.2. Protein purification   

Complex IV was accidentally copurified from mitochondrial membranes of *N. crassa* (Künkele *et al.*, 1998 ▸; Ahting *et al.*, 1999 ▸) by immobilized metal-affinity chromatography (IMAC) without introducing an affinity tag, most likely on account of the internal histidine-rich region in the Cox4 subunit (Supplementary Fig. S3). Isolated *N. crassa* mitochondria (2 g) were solubilized in 20% glycerol, 20 m*M* Tris pH 8.5, 300 m*M* NaCl, 20 m*M* imidazole, 1%(*w*/*v*) DDM and four tablets of cOmplete (EDTA-free) protease inhibitor (Roche) for 1 h at 4°C. The solution was centrifuged at 150 000*g* for 40 min at 4°C and the resulting supernatant was incubated with 10 ml Nickel-complexed Chelating Sepharose (GE Healthcare) for 30 min. The resin was washed with 10% glycerol, 50 m*M* HEPES pH 7.2, 0.1%(*w*/*v*) DDM, 1 m*M* PMSF. The protein was eluted with 300 m*M* imidazole in the same buffer. The protein concentration of the yellow protein fraction was 1.9 mg ml^−1^. A 5 ml volume was used for reconstitution into nanodiscs with the membrane-scaffold protein MSP2N2.

### 2.3. Membrane-scaffold protein purification   

The gene for MSP2N2 was cloned into a pET-28a vector with an N-terminal His tag and a TEV cleavage site. The protein was expressed in *Escherichia coli* BL21(DE3) cells for 3 h in TB medium at 37°C after induction with IPTG. The cells were disrupted in 50 m*M* Tris pH 8, 150 m*M* NaCl, 1 m*M* PMSF, 1% Triton X-100. After high-speed centrifugation, the supernatant was incubated with Nickel-complexed Chelating Sepharose (GE Healthcare) and washed in three steps with 40 m*M* Tris pH 8, 300 m*M* NaCl, 1% Triton X-100, then with 40 m*M* Tris pH 8, 300 m*M* NaCl, 50 m*M* sodium cholate, 20 m*M* imidazole and finally with 40 m*M* Tris pH 8, 300 m*M* NaCl, 40 m*M* imidazole. MSP2N2 was purified by IMAC using the N-terminal His tag. The protein was eluted with 40 m*M* Tris pH 8, 300 m*M* NaCl, 400 m*M* imidazole. After dialysis against 20 m*M* Tris pH 8, 100 m*M* NaCl overnight, the His tag was cleaved off using TEV protease and separated from the cleaved MSP2N2 by a second IMAC step (His-Select, Sigma) using the unbound flowthrough.

### 2.4. Reconstitution of complex IV into lipid nanodiscs   

For reconstitution, 950 µl of lipids [4 m*M* POPC, 3.5 m*M* POPE, 2.5 m*M* cholesterol in 0.5%(*w*/*v*) DDM] and 1.97 ml MSP2N2 (4 mg ml^−1^) were added to the eluted mitochondrial protein, resulting in a final molar protein:MSP2N2:lipids ratio of 1:3:150. 7.5 ml of the mixture was incubated for 1 h at room temperature before the detergent was removed by incubation with 500 mg BioBeads SM-2 (Bio-Rad) for 2.5 h. The solution was dialyzed against 20 m*M* HEPES pH 7.2, 20 m*M* KCl, 5 m*M* β-mercaptoethanol overnight. The sample was centrifuged for 5 min to remove aggregated protein and concentrated to a final volume of 300 µl using a 100 kDa cutoff Amicon centrifugation tube (Merck). 50 µl of sample was applied onto a Superose 6 Increase 3.2/300 gel-filtration column (GE Healthcare) and yellow complex IV fractions were collected for cryo-EM sample preparation.

### 2.5. Cryo-EM data acquisition   

**Figure 1:**
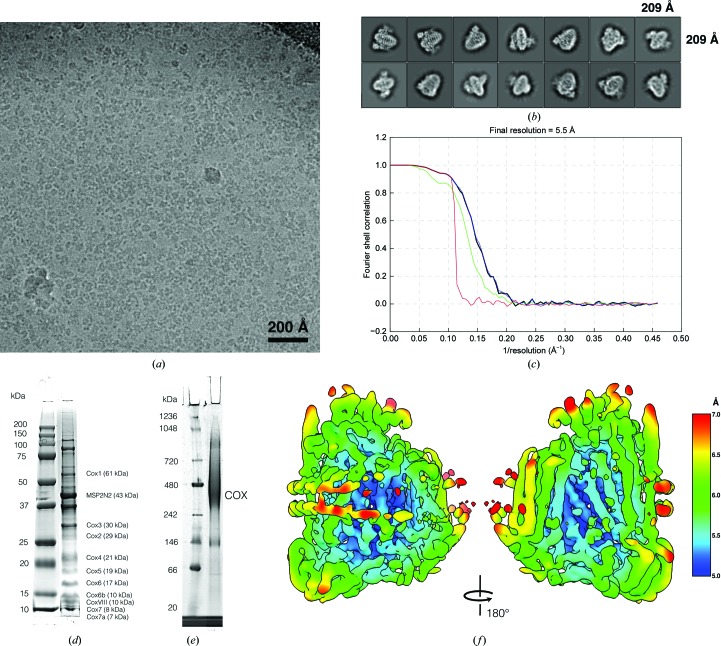
Data acquisition and image processing. (*a*) Typical electron micrograph as used for particle picking and image processing with *RELION*-3. (*b*) 2D class averages of complex IV with bound hemes visible as a single bright dot in the first two averages. (*c*) Fourier shell correlation and 0.143 cutoff. Refinement resulted in a map resolution of 5.5 Å. Black, corrected FSC; blue, masked FSC; green, unmasked FSC; red, phase-randomized FSC. All images were displayed within *RELION*-3. (*d*) SDS–PAGE of the fraction used for cryo-EM. (*e*) The same fraction on a blue native PAGE. Complex IV labeled as COX. (*f*) Local resolution map from *RELION*-3.

The purified protein–nanodisc complex was applied onto C-Flat 1/1 grids (400 mesh) and plunge-frozen in a Vitrobot (ThermoFisher) with 7 s blotting. Data were automatically acquired at 300 kV with *Leginon* (Suloway *et al.*, 2005 ▸) in a Polara electron microscope (FEI) equipped with an energy filter (slit width of 70 eV) and a K2 direct electron detector (Gatan) in counting mode. A total of 3467 micrographs were recorded, of which 2835 were selected manually for further processing. A typical micrograph is shown in Fig. 1 ▸(*a*). Data-acquisition and processing parameters are summarized in Table 1 ▸.

### 2.6. Image processing   

Micrographs were processed with *RELION*-3 (Zivanov *et al.*, 2018 ▸). Particles were picked automatically and extracted with a box size of 192 × 192 pixels. Subsequent classification in 2D (100 classes) and selection resulted in 2 418 748 particles with no obvious ice contamination or carbon features. Particles were 3D-classified into six classes, with a first model generated *ab initio* in *RELION*-3 using 109 979 particles from ten selected 2D class averages as a reference [see Fig. 1 ▸(*b*)]. 1 656 842 selected particles were classified again into six classes, resulting in one single, homogeneous class with 426 592 particles that were refined by gold-standard auto-refinement in *RELION*-3 to 7 Å resolution. Bayesian polishing was applied to the refined particles (Zivanov *et al.*, 2019 ▸). The polished particles were classified into three classes, resulting in 134 734 particles (31%) with lower noise levels, clearer protein features and a homogeneous distribution of orientations. A final 3D refinement step resulted in a map with a gold-standard resolution of 5.5 Å [FSC cutoff at 0.143; see Fig. 1 ▸(*c*)]. The final map was sharpened with a *B* factor of −370 Å^2^. The map has been deposited in the EMDB under code EMD-4720.

### 2.7. Model fitting and structure comparison   

**Figure 2:**
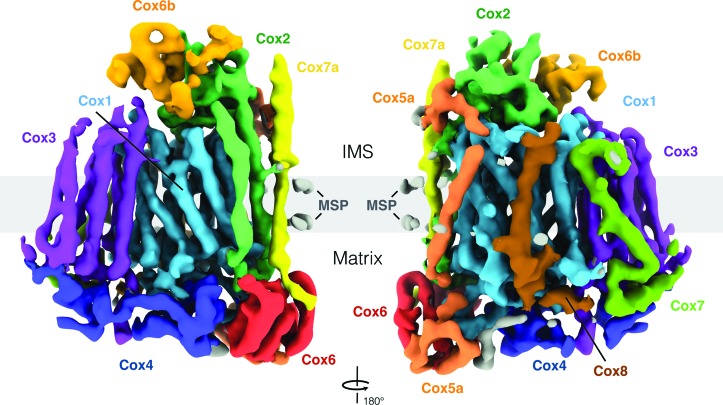
5.5 Å resolution cryo-EM map of *N. crassa* complex IV (σ level 0.02). Subunits are individually colored and assigned according to complex IV in the *S. cerevisiae* supercomplex (PDB entry 6hu9). Other densities are in gray.

**Figure 3:**
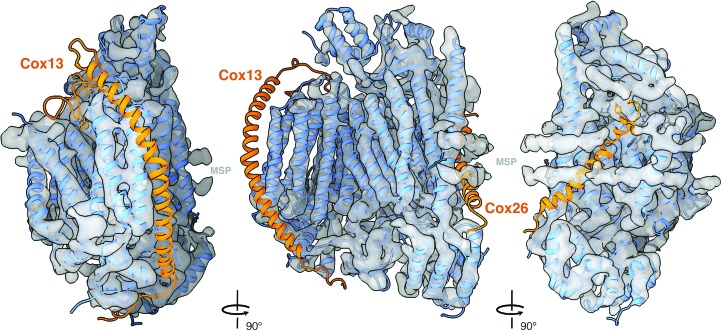
Comparison of complex IV from *N. crassa* and *S. cerevisiae*. The *S. cerevisiae* model (PDB entry 6hu9) was fitted into the *N. crassa* cryo-EM map. Chains that fit the density map are drawn as blue ribbons. Ribbon diagrams of the Cox13 and Cox26 subunits that are not present in the *N. crassa* structure are colored orange.

**Figure 4:**
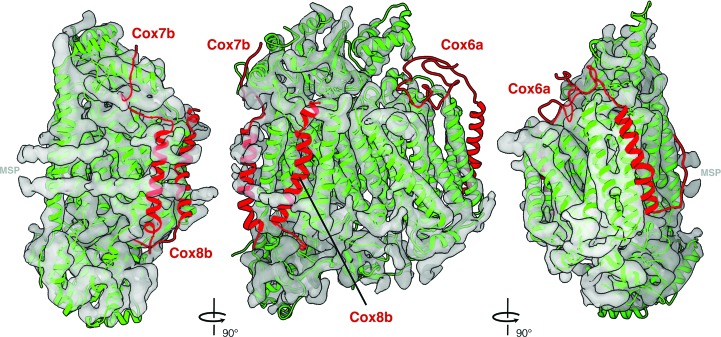
Comparison of complex IV from *N. crassa* and *B. taurus*. Subunits of the *B. taurus* model (PDB entry 1occ) that fit the *N. crassa* map are shown in green. The Cox6a, Cox7b and Cox8b subunits of the bovine model (red) are absent in the *N. crassa* map.

Atomic models of complex IV from *S. cerevisiae* (PDB entry 6hu9, chains *a*–*l*) and *Bos taurus* (PDB entry 1occ, chains *A*–*M*) were fitted into the density by rigid-body docking in *UCSF Chimera* (Pettersen *et al.*, 2004 ▸; Figs. 3 and 4). The two models were converted to a simulated map at 5.5 Å resolution before fitting. The correlation values were 0.78 for the *S. cerevisiae* model and 0.72 for the *B. taurus* model. The *N. crassa* map density was colored by subunit (*S. cerevisiae*) and visualized with *ChimeraX* (Goddard *et al.*, 2018 ▸; Fig. 2). For comparison, homologous protein sequences (from UniProtKB) of *N. crassa* complex IV subunits were aligned with the *S. cerevisiae* subunits using *Clustal Omega* 1.2.4 (Sievers & Higgins, 2014 ▸; see Supplementary Figs. S1–S5).

## 3. Results and discussion   

### 3.1. Overall structure   

The complex that was accidentally copurified from *N. crassa* mitochondria was identified as complex IV by cryo-EM and image processing. The 5.5 Å resolution map showed clear density for ten different complex IV subunits and the membrane-scaffold protein (Fig. 2 ▸). The transmembrane core subunit Cox1 has 12 transmembrane helices (see also Supplementary Fig. S1) surrounding two strong, globular densities for the two bound hemes that were already visible in 2D class averages [Fig. 1 ▸(*b*)]. The second core subunit Cox2 consists of two transmembrane helices (Supplementary Fig. S2) and a large domain in the intermembrane space (IMS). With seven transmembrane helices (Supplementary Fig. S2) Cox3 is the second-largest subunit, with one loop protruding into the matrix. Cox4, Cox6 and Cox6b do not have transmembrane helices (Supplementary Fig. S3) and are either attached on the IMS or the matrix side of the complex. Cox4 and Cox6 are the major soluble domains on the matrix side. Cox6b is located in the IMS and interacts tightly with the soluble domain of Cox2. The small subunits Cox5a, Cox7, Cox8 and Cox7a surround the larger core subunits with single transmembrane spans (Supplementary Fig. S4). Cox5a has domains on both the matrix and the IMS sides.

### 3.2. Comparison with complex IV structures from *S. cerevisiae* and *B. taurus*   

The structure of *N. crassa* complex IV is very similar to that from *S. cerevisiae*, which was purified as part of a supercomplex (Hartley *et al.*, 2019 ▸; Rathore *et al.*, 2019 ▸). The atomic model of the yeast complex (PDB entry 6hu9) fitted the cryo-EM density without adjustment. The *N. crassa* complex was present in lipid nanodiscs in one single conformation. Complexes of different subunit compositions were not identified in the 3D classes, indicating that the lipid environment did not result in significant structural differences compared with complex IV in the *S. cerevisiae* supercomplex. The membrane-scaffold protein (MSP) was easily visible as a double belt around the transmembrane helices (Figs. 2 ▸ and 3 ▸), which rules out the presence of additional subunits in the *N. crassa* complex. As in other cryo-EM structures of membrane-protein complexes (Hahn *et al.*, 2018 ▸), a single scaffold protein chain wraps around the assembly twice. In our case, it is roughly twice as long as the circumference of complex IV.

The *N. crassa* complex IV has only ten subunits, two fewer than that from *S. cerevisiae*. As shown in Fig. 3 ▸, our map had no densities for *S. cerevisiae* subunit Cox13 or the recently identified subunit Cox26 (Levchenko *et al.*, 2016 ▸). The *N. crassa* genome contains a homologous sequence for Cox13 but not for Cox26 (Supplementary Fig. S5), which is therefore absent, as is also the case in the bovine complex (Luo *et al.*, 2017 ▸; Fig. 4 ▸). Cox6a in the bovine structure is homologous to Cox13 and is found in an analogous location, but its structure is slightly different. In *S. cerevisiae* it is present in one supercomplex structure (Hartley *et al.*, 2019 ▸) but not in the other (Rathore *et al.*, 2019 ▸), suggesting a possible regulatory role. Cox13 may be involved in complex IV dimer formation and might not always be expressed (Hartley *et al.*, 2019 ▸). This would explain its absence in our map of the isolated monomer.

The crystal structure of *B. taurus* complex IV (PDB entry 1occ; Tsukihara *et al.*, 1996 ▸) fits our map less closely. Three subunits of the bovine crystal structure, Cox6a, Cox7b and Cox8b, do not fit into the *N. crassa* map (Fig. 4 ▸). Cox7b and Cox8b are absent in the *S. cerevisiae* model, indicating that they are not needed in fungi. The *N. crassa* structure therefore shows the ten core subunits of complex IV that are present in most, if not all, other eukaryotes and appear to be a minimal set. It is safe to assume that the function of the central subunits Cox1, Cox2 and Cox3 in catalyzing electron transfer and proton pumping is identical in *S. cerevisiae*, *N. crassa* and mammals. Presumably, the seven other subunits are necessary for complex IV assembly or supercomplex formation. It will be interesting to investigate whether some of these subunits mediate interaction with complex I in the *N. crassa* supercomplex, as in mammalian respirasomes.

**Figure 5:**
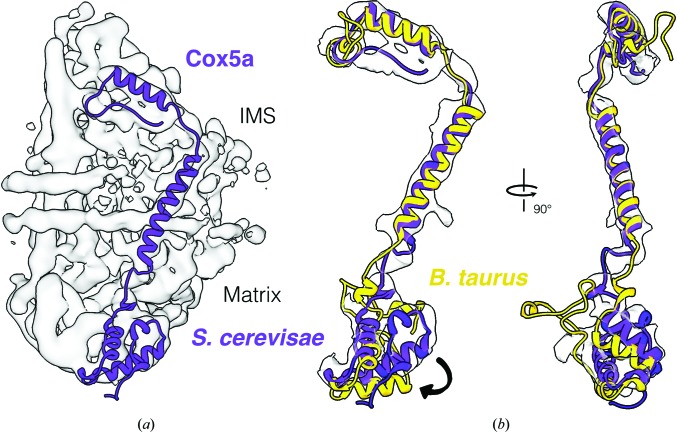
Comparison of the Cox5a subunit from *S. cerevisiae* (purple) and *B. taurus* (yellow) fitted to the *N. crassa* map. (*a*) *S. cerevisiae* Cox5a. (*b*) Overlay of fitted Cox5a from *B. taurus* and *S. cerevisiae*. The observed structural difference in the matrix domain is indicated by an arrow.

A respiratory supercomplex for *N. crassa* has been described biochemically (Marques *et al.*, 2007 ▸), whereas cryo-EM maps have been determined for the *S. cerevisiae* and *B. taurus* supercomplexes (Hartley *et al.*, 2019 ▸; Rathore *et al.*, 2019 ▸; Sousa *et al.*, 2016 ▸; Gu *et al.*, 2016 ▸). A major difference in the structures of complex IV is visible in the matrix domain of Cox5a. It has been suggested that this domain mediates interaction with complex III in the *S. cerevisiae* supercomplex (Hartley *et al.*, 2019 ▸). The overall interaction between complex III and complex IV in the *S. cerevisiae* supercomplex is remarkably different from that in the bovine supercomplex, possibly because of a difference in the structure of Cox5a. Since Cox5a of yeast and *N. crassa* have the same structure (Fig. 5 ▸) and the *N. crassa* complex IV is unassociated and incorporated into lipid nanodiscs, the observed difference is unlikely to be owing to an interaction with complex III. However, such an interaction is likely to occur in the *N. crassa* supercomplex.

Another striking difference in the *N. crassa* structure compared with that from *S. cerevisiae* is an additional rod-shaped density between the matrix domains of Cox4 and Cox5a (gray density in Fig. 2 ▸). Since the sequence of *N. crassa* Cox4 is longer by 29 residues than that from *S. cerevisiae* (Supplementary Fig. S3), we suggest that this density belongs to the C-terminal tail of *N. crassa* Cox4, although at 5.5 Å resolution we cannot rule out that it belongs to another subunit. The density may be unique to *N. crassa*, as it is also absent in bovine complex IV (Fig. 4 ▸).

## 4. Conclusion   

With recent technological and computational developments (Kühlbrandt, 2014 ▸; McMullan *et al.*, 2016 ▸; Elmlund *et al.*, 2017 ▸; Zivanov *et al.*, 2018 ▸), cryo-EM is developing into a method that can identify protein complexes of suitable size in heterogenous preparations quickly and unambiguously: in our case, *N. crassa* complex IV that was inadvertently copurified by nickel-affinity chromatography was identified by its similarity to the known structures of the *S. cerevisiae* and *B. taurus* complexes. It is particularly similar to that from *S. cerevisiae*, even though it lacks two of its subunits. The ten core subunits of the *N. crassa* complex are present in both the *S. cerevisiae* and *B. taurus* complexes. The major difference compared with the *B. taurus* complex is the structure of the matrix domain of the Cox5a subunit, which appears to be typical for a fungal complex IV. The subunit may mediate contact with complex III in a supercomplex. Unlike *S. cerevisiae*, *N. crassa* has complex I and may therefore form a supercomplex that combines features of the *S. cerevisiae* and mammalian supercomplexes. The major difference of the *N. crassa* complex IV is the density between the Cox4 and Cox5a subunits, which presumably belongs to the histidine-rich C-terminus of Cox4. The functional role of this feature is unknown.

Although the structures of *N. crassa* and *S. cerevisiae* complex IV are largely identical, it would be interesting to investigate the structure of a fungal supercomplex that includes complex I, which is absent in *S. cerevisiae* (Sousa & Vonck, 2019 ▸) but is present in *N. crassa*. The *N. crassa* supercomplex may be more similar to the mammalian respirasome than to the *S. cerevisiae* supercomplex.

## Supplementary Material

Supplementary Figures (sequence alignments). DOI: 10.1107/S2052252519007486/pw5006sup1.pdf

## Figures and Tables

**Table 1:** Parameters for electron-microscopy data acquisition, image processing and model docking

Data acquisition	
Electron microscope	FEI Polara
Direct electron detector	K2 Summit (Gatan)
Software	*Leginon*
Magnification	200 000
Voltage (kV)	300
Energy-filter slit width (eV)	70
Micrographs collected	3467
Micrographs used	2835
Frames per exposure	40
Exposure time (s)	8
Per-frame electron exposure (e^–^ Å^−2^)	1.5
Defocus range (µm)	−0.8 to −4.5
Pixel size (Å)	1.09
Processing	
Software	*RELION*-3
Initial particle images	2418748
Extraction box size (pixels)	192 × 192
Initial model	*Ab initio*
Symmetry imposed	*C*1
Final particle images	134734
Refinement type	Gold standard
Map resolution (Å)	5.5
FSC threshold	0.143
Map-sharpening *B* factor (Å^2^)	−370
Map resolution range (Å)	5.0–7.0
Rigid-body docking	
Software	*UCSF Chimera*
Cross-correlation, PDB entry 6hu9 (chains *a*–*l*)	0.78
Cross-correlation, PDB entry 1occ (chains *A*–*M*)	0.72
