# Supplementary material for: Cryo-EM structure of Neurospora crassa respiratory complex IV
Source: IUCrJ. 2019 Jun 26;6(Pt 4):773–80. doi: 10.1107/S2052252519007486 (PMC6608615; doi:10.1107/S2052252519007486)
Supplement: Supplementary file 1 [file m-06-00773-sup1.pdf]

IUCrJ

Volume 6 (2019)

Supporting information for article:

Cryo-EM structure of *Neurospora crassa* respiratory complex IV

Thomas Bausewein, Stephan Nussberger and Werner Kühlbrandt

N. crassa Cytochrome c oxidase subunit 1 (named Cox1), mitochondrially encoded
61.17 % identity with _S. cerevisiae_ Cytochrome c oxidase subunit 1 (chain a in PDB 6HU9)

```
SP|P03945|COX1_NEUCR MSSISIWTERWFLSTNAKDIGVLYLIFALFSGLLGTAFSVLIRMELSGPGVQYIAD-NQL 59
SP|P00401|COX1_YEAST ------MVQRWLYSTNAKDIAVLYFMLAIFSGMAGTAMSLIIRLELAAPGSQYLHGNSQL 54
                            .:**: *******.***:::*:***: ***:*::**:**:.** **: . .**

SP|P03945|COX1_NEUCR YNAIITAHAILMIFFMVMPALIGGFGNFLLPLLVGGPDMAFPRLNNISFWLLPPSLLLLV 119
SP|P00401|COX1_YEAST FNVLVVGHAVLMIFFLVMPALIGGFGNYLLPLMIGATDTAFPRINNIAFWVLPMGLVCLV 114
                     :*.::..**:*****:***********:****::*. * ****:***:**:** .*: **

SP|P03945|COX1_NEUCR FSACIEGGAGTGWTIYPPLSGVQSHSGPSVDLAIFALHLSGVSSLLGSINFITTIVNMRT 179
SP|P00401|COX1_YEAST TSTLVESGAGTGWTVYPPLSSIQAHSGPSVDLAIFALHLTSISSLLGAINFIVTTLNMRT 174
                      *: :*.*******:*****.:*:***************:.:*****:****.* :****

SP|P03945|COX1_NEUCR PGIRLHKLALFGWAVVITAVLLLLSLPVLAGAITMLLTDRNFNTSFFETAGGGDPILFQH 239
SP|P00401|COX1_YEAST NGMTMHKLPLFVWSIFITAFLLLLSLPVLSAGITMLLLDRNFNTSFFEVSGGGDPILYEH 234
                      *: :*** ** *::.***.*********:..***** **********.:*******::*

SP|P03945|COX1_NEUCR LFWFFGHPEVYILIIPGFGIISTTISAYSNKSVFGYIGMVYAMMSIGILGFIVWSHHMYT 299
SP|P00401|COX1_YEAST LFWFFGHPEVYILIIPGFGIISHVVSTYSKKPVFGEISMVYAMASIGLLGFLVWSHHMYI 294
                     ********************** .:*:**:* *** *.***** ***:***:*******

SP|P03945|COX1_NEUCR VGLDVDTRAYFTAATLIIAVPTGIKIFSWLATCYGGSIRLTPSMLFALGFVFMFTIGGLS 359
SP|P00401|COX1_YEAST VGLDADTRAYFTSATMIIAIPTGIKIFSWLATIHGGSIRLATPMLYAIAFLFLFTMGGLT 354
                     ****.*******:**:***:************ :******:  **:*:.*:*:**:***:

SP|P03945|COX1_NEUCR GVVLANASLDIAFHDTYYVVAHFHYVLSMGAVFAMFSGWYHWVPKILGLNYNMVLSKAQF 419
SP|P00401|COX1_YEAST GVALANASLDVAFHDTYYVVGHFHYVLSMGAIFSLFAGYYYWSPQILGLNYNEKLAQIQF 414
                     **.*******:*********.**********:*::*:*:*:* *:*******  *:: **

SP|P03945|COX1_NEUCR WLLFIGVNLTFFPQHFLGLQGMPRRISDYPDAFSGWNLISSFGSIVSVVASWLFLYIVYI 479
SP|P00401|COX1_YEAST WLIFIGANVIFFPMHFLGINGMPRRIPDYPDAFAGWNYVASIGSFIATLSLFLFIYILYD 474
                     **:***.*: *** ****::****** ******:*** ::*:**:::.:: :**:**:*

SP|P03945|COX1_NEUCR QLVQGEYAGRYPWS-----IPQFYT-DSLRALLNRSYPSLEWSISSPPKPHSFVSLPLQS 533
SP|P00401|COX1_YEAST QLVNGLNNKVNNKSVIYNKAPDFVESNTIFNLNTVKSSSIEFLLTSPPAVHSFNTPAVQS 534
                     ***:*        *      *:*   :::  * . .  *:*: ::***  *** :  :**

SP|P03945|COX1_NEUCR SSFFLSFFRLSSYGEQKEISGRQN 557
SP|P00401|COX1_YEAST ------------------------
```

Figure S1 Sequence alignment of Cox1 from *N. crassa* and *S. cerevisiae* with Clustal Omega (1.2.4). Bold letters show transmembrane helices visible in the map. *N. crassa* has a longer C-terminus than *S. cerevisiae*.

N. crassa Cytochrome c oxidase subunit 2 (named Cox2), mitochondrially encoded
57.087 % identity with _S. cerevisiae_ Cytochrome c oxidase subunit 2 (chain b in PDB 6HU9)

```
SP|P00411|COX2_NEUCR ----MGLLFNNLIMNFDAPSPWGIYFQDSATPQMEGLVELHDNIMYYLVVILFGVGWILL 56
SP|P00410|COX2_YEAST MLDLLRLQLTTF-IMNDVPTPYACYFQDSATPNQEGILELHDNIMFYLLVILGLVSWMLY 59
                         : * :..: :  *.*:*:. ********: **::*******:**:***  *.*:*

SP|P00411|COX2_NEUCR SIIRNYISTKSPISHKYLNHGTLIELIWTITPAVILILIAFPSFKLLYLMDEVSDPSMSV 116
SP|P00410|COX2_YEAST TIVMTY--SKNPIAYKYIKHGQTIEVIWTIFPAVILLIIAFPSFILLYLCDEVISPAMTI 117
                     :*: .*  :*.**::**::**  **:**** *****::****** **** *** .*:*::

SP|P00411|COX2_NEUCR LAEGHQWYWSYQYPDFLDSNDEFIEFDSYIVPESDLEEGALRMLEVDNRVILPELTHVRF 176
SP|P00410|COX2_YEAST KAIGYQWYWKYEYSDFINDSGETVEFESYVIPDELLEEGQLRLLDTDTSMVVPVDTHIRF 177
                      * *:****.*:* **::...* :**:**::*:. **** **:*:.*. :::*  **:**

SP|P00411|COX2_NEUCR IITAGDVIHSFAVPSLGVKCDAYPGRLNQVSVFINREGVFYGQCSEICGILHSSMPIVIE 236
SP|P00410|COX2_YEAST VVTAADVIHDFAIPSLGIKVDATPGRLNQVSALIQREGVFYGACSELCGTGHANMPIKIE 237
                     ::**.****.**:****:* ** ********.:*:******* ***:**  *:.*** **

SP|P00411|COX2_NEUCR SVSLEKFLTWLEEQ 250
SP|P00410|COX2_YEAST AVSLPKFLEWLNEQ 251
                     :*** *** **:**
```

N. crassa Cytochrome c oxidase subunit 3 (named Cox3), mitochondrially encoded
53.532 % identity with _S. cerevisiae_ Cytochrome c oxidase subunit 3 (chain c in PDB 6HU9)

```
SP|P00422|COX3_NEUCR MTNLIRSNFQDHPFHLVSPSPWPLNTSVCLLNLTTTGALSMHNFNNIHYLYYIALIGLVS 60
SP|P00420|COX3_YEAST MTHLERSRHQQHPFHMVMPSPWPIVVSFALLSLALSTALTMHGYIGNMNMVYLALFVLLT 60
                     **:* **..*:****:* *****: .*..**.*: : **:**.: .   : *:**: *::

SP|P00422|COX3_NEUCR AMFLWFRDIISEGTFLGDHTLAVQRGLNLGIILFIVSEALFFLAIFWAFFHSALTPTVEL 120
SP|P00420|COX3_YEAST SSILWFRDIVAEATYLGDHTMAVRKGINLGFLMFVLSEVLIFAGLFWAYFHSAMSPDVTL 120
                     : :******::*.*:*****:**::*:***:::*::**.*:* .:***:****::* * *

SP|P00422|COX3_NEUCR GAQWPPIGIEPVNPFELPLLNTVILLSSGATITYAHHALIKGEREGALYGSIATILLAII 180
SP|P00420|COX3_YEAST GACWPPVGIEAVQPTELPLLNTIILLSSGATVTYSHHALIAGNRNKALSGLLITFWLIVI 180
                     ** ***:*** *:* *******:********:**:***** *:*: ** * : *: * :*

SP|P00422|COX3_NEUCR FTGFQGVEYSVSSFTISDGAFGTCFFFSTGFHGIHVIIGTIFLAVALWRIFAYHLTDNHH 240
SP|P00420|COX3_YEAST FVTCQYIEYTNAAFTISDGVYGSVFYAGTGLHFLHMVMLAAMLGVNYWRMRNYHLTAGHH 240
                     *.  * :**: ::******.:*: *: .**:* :*::: : :*.*  **:  **** .**

SP|P00422|COX3_NEUCR VGFEGGILYWHFVDVVWLFLYISVYYWGS 269
SP|P00420|COX3_YEAST VGYETTIIYTHVLDVIWLFLYVVFYWWGV 269
                     **:*  *:* *.:**:*****: .*:**
```

Figure S2 Sequence alignment of *N. crassa* and *S. cerevisiae* Cox2 and Cox3 with Clustal Omega (1.2.4). Bold letters show transmembrane helices visible in the map, grey letters show amino acids absent in the *S. cerevisiae* model (PDB 6HU9).

N. crassa Cytochrome c oxidase subunit 4 (named Cox4), nuclear-encoded
41.176 % identity with _S. cerevisiae_ Cytochrome c oxidase subunit 4 (chain d in PDB 6HU9)

```
SP|P06809|COX4_NEUCR MLLSRTAVAVARRATAAPALRRSIATTVVRCNAETK-PVPHIKRLSEIKTKDDLFGPGAA 59
SP|P04037|COX4_YEAST MLSLRQSIRFFKPAT-----RT-LC--SSRYLLQQKPVVKTAQNLAEVNGPETLIGPGAK 52
                     **  * :: . : **     *  :.    *   : *  *   :.*:*::  : *:****

SP|P06809|COX4_NEUCR PGTVPTDLEQATGLERLEILGKMEGVDVFDMKPLDASRRGTMENPISVRSAGDEQYAGCT 119
SP|P04037|COX4_YEAST EGTVPTDLDQETGLARLELLGKLEGIDVFDTKPLDSSRKGTMKDPIIIESYDDYRYVGCT 112
                      *******:* *** ***:***:**:**** ****:**:***::** :.* .* :*.***

SP|P06809|COX4_NEUCR GFPADSHNVIWLTMTRERPVERCPECGNVYKMDYVGPQDDHAHDHGHDHHGFEEPKTFAD 179
SP|P04037|COX4_YEAST GSPAGSHTIMWLKPTV-NEVARCWECGSVYKLNPVGVPNDDHHH---------------- 155
                     * **.**.::**. *  . * ** ***.***:: **  :*. *.

SP|P06809|COX4_NEUCR YVKPEYW 186
SP|P04037|COX4_YEAST -------
```

N. crassa Cytochrome c oxidase subunit 6 (named Cox6), nuclear-encoded
51.656 % identity with _S. cerevisiae_ Cytochrome c oxidase subunit 6 (chain f in PDB 6HU9)

```
SP|Q01359|COX6_NEUCR MASFFRTAVRGPSAGLFRAVARPQP---IAARVSLFSTSSRFRSEHHEETFEEFTARYEK 57
SP|P00427|COX6_YEAST --MLSRAIFRNPVINRTLLRARPGAYHATRLTKNTFIQSRKYSDAHDEETFEEFTARYEK 58
                        : *: .*.*  .     ***          . *  * :: . *.*************

SP|Q01359|COX6_NEUCR EFDAVQDVFELQRNLNNAFAYDLVPSPSVLAAALKAARRVNDFPTAVRVFEGIKAKVENK 117
SP|P00427|COX6_YEAST EFDEAYDLFEVQRVLNNCFSYDLVPAPAVIEKALRAARRVNDLPTAIRVFEALKYKVENE 118
                     *** . *:**:** ***.*:*****:*:*:  **:*******:***:****.:* ****:

SP|Q01359|COX6_NEUCR GQYEQYLAELKPLREELGITLKEDLYPEEAN 148
SP|P00427|COX6_YEAST DQYKAYLDELKDVRQELGVPLKEELFPSSS- 148
                     .**: ** *** :*:***: ***:*:*..:
```

N. crassa Cytochrome c oxidase subunit (named Cox6b), nuclear-encoded
53.488 % identity with _S. cerevisiae_ Cytochrome c oxidase subunit 6B (chain j in PDB 6HU9)

```
TR|Q1K8U2|Q1K8U2_NEUCR MSDDERVTKPFKFVTGVDARFPNVNQTKHCWQNYVDYHKCILAKGEDFAPCRQFWLAYRS 60
SP|Q01519|COX12_YEAST  MADQEN---SPLHTVGFDARFPQQNQTKHCWQSYVDYHKCVNMKGEDFAPCKVFWKTYNA 57
                       *:*:*.      ...*.*****: ********.*******:  ********: ** :*.:

TR|Q1K8U2|Q1K8U2_NEUCR LCPSGWYQRWDEQREAGNFPVKLE-- 84
SP|Q01519|COX12_YEAST  LCPLDWIEKWDDQREKGIFAGDINSD 83
                       *** .* ::**:*** * *  .::
```

Figure S3 Sequence alignments of soluble subunits Cox4, Cox6 and Cox6b from *N. crassa* and *S. cerevisiae* with Clustal Omega (1.2.4). Grey letters indicate residues that are absent in the *S. cerevisiae* model (PDB 6HU9).

N. crassa Cytochrome c oxidase polypeptide 5 (named Cox5a), nuclear-encoded
29.31 % identity with _S. cerevisiae_ Cytochrome c oxidase polypeptide 5a (chain e in PDB 6HU9)

```
SP|P06810|COX5_NEUCR  MLRTPTVSALVRNVAVRAA--KPTMAVRAASTMPISNPTLANIEKRWEQMPMQEQAELWM 58
SP|P00424|COX5A_YEAST ---------MLRNTFTRAGGLSRITSVRFAQTHALSNAAVMDLQSRWENMPSTEQQDIVS 51
                               ::**. .**.  .    :** *.*  :** :: :::.***:**  ** ::

SP|P06810|COX5_NEUCR  ALRDRMKGNWADLTLQEKKAAYYIAFGPHGPRALPPP-GEQKKVLAYTVAGVFLSFVIFA 117
SP|P00424|COX5A_YEAST KLSERQKLPWAQLTEPEKQAVWYISYGEWGPRRPVLNKGDSSFIAKGVAAGLLFSVGLFA 111
                        * :* *  **:**  **:*.:**::*  ***      *:.. :   ..**:::*. :**

SP|P06810|COX5_NEUCR  TMRAFAKPPPATMTKEWQEATNEFLKAQKSDPLTGLTSEGYNGKGHVQSPSASA 171
SP|P00424|COX5A_YEAST VVRMAGGQDAKTMNKEWQLKSDEYLKSKNANPWGGYSQVQSK------------ 153
                      .:*  .     **.****  ::*:**:::::*  * :.   :
```

N. crassa Uncharacterized protein (named Cox7), nuclear-encoded
15.493 % identity with _S. cerevisiae_ Cytochrome c oxidase subunit 7 (chain g in PDB 6HU9)

```
TR|Q7S7U5|Q7S7U5_NEUCR MPGLVNAPNHVPEKQRYYQQAFKNHTRLWKIGPRSGIIMTTFNIAMWGTFGASMYAMSRK 60
SP|P10174|COX7_YEAST   ------MANKVIQLQKIFQS---STKPLWWRHPRSALYLYPFYAIFAVAVVTPLLYIPNA 51
                              *:* : *: :*.   . . **   ***.: :  *   :  :. : :  : .

TR|Q7S7U5|Q7S7U5_NEUCR VLGYNTWFSED 71
SP|P10174|COX7_YEAST   IRGIKAKKA-- 60
                       : * ::  :
```

N. crassa Cytochrome c oxidase chain VIIc (named Cox8), nuclear-encoded
16.471 % identity with _S. cerevisiae_ Cytochrome c oxidase polypeptide VIII (chain h in PDB 6HU9)

```
TR|Q1K528|Q1K528_NEUCR MFSRVALRAAPRQQPFSLVARRTFQTTRAQLSSPYHYPEGPRSNLPFNPKTRFFWFRY-- 58
SP|P04039|COX8_YEAST   MLCQQMIRTTAKRS-------SNIMTRPIIMKRSVHFKDGVYENIPFKVKGRKTPYALSH 53
                       *:.:  :*:: ::.        .: *    :.   *: :*  .*:**: * *    :

TR|Q1K528|Q1K528_NEUCR LMYCVVGFGSPVAIAVWQTYRPRS- 82
SP|P04039|COX8_YEAST   FGFFAIGFAVPFVACYVQLKKSGAF 78
                       : : .:**. *.. .   *  :  :
```

N. crassa Cytochrome c oxidase subunit 7A (named Cox7a), nuclear-encoded
32.812 % identity with _S. cerevisiae_ Cytochrome c oxidase subunit 7A (chain i in PDB 6HU9)

```
TR|Q7S5M7|Q7S5M7_NEUCR MAATAVRPITGMLRRGLILDIGIALGVGFVMANGYWYGYHMPRTNARDNYYKKLEEERAA 60
SP|P07255|COX9_YEAST   ---MTIAPITGTIKRRVIMDIVLGFSLGGVMASYWWWGFHMDKINKREKFYAELAERKKQ 57
                           :: **** ::* :*:** :.:.:* ***. :*:*:** : * *:::* :* *.:

TR|Q7S5M7|Q7S5M7_NEUCR RMGA 64
SP|P07255|COX9_YEAST   EN-- 59
                       .
```

Figure S4 Sequence alignments of *N. crassa* and *S. cerevisiae* subunits Cox5a, Cox7, Cox8 and Cox7a with Clustal Omega (1.2.4). Grey letters indicate residues that are absent in the *S. cerevisiae* model (PDB 6HU9), bold letters mark transmembrane regions. All proteins have one transmembrane helices.

N. crassa Encodes anonymous transcript-5 protein (named Cox6a), nuclear-encoded
33.813 % identity with _S. cerevisiae_ Cytochrome c oxidase subunit 6A, named Cox13 (chain k in PDB 6HU9)

```
TR|V5IRD7|V5IRD7_NEUCR MFAQRQMFFARLAANLRAPAVRQTVQRRFASTPANESGKNAFVREREAVKQHAAETTELW 60
SP|P32799|COX13_YEAST  MFRQCAKRY---ASSLPPN----ALKP--AFGPPDKVAAQKFKESLMATEKHAKDTSNMW 51
                       ** *    :   *:.*       :::   *  * :: . : * ..  *.::** :*:::*

TR|V5IRD7|V5IRD7_NEUCR RKISLYGIPPALALAGYNAYTLYNEHWE---HWSHLPPLEERTEYPYQNIRTRNYPWGDG 117
SP|P32799|COX13_YEAST  VKISVWVALPAIALTAVNTYFVEKEHAEHREHLKHVPDSEWPRDYEFMNIRSKPFFWGDG 111
                        ***::   **:**:. *:* : :** *   * .*:*  *   :* : ***:: : ****

TR|V5IRD7|V5IRD7_NEUCR DKTLFWNESVNYHNRDKVT 136
SP|P32799|COX13_YEAST  DKTLFWNPVVNRHIEHDD- 129
                       *******  ** * ...
```

S. cerevisiae Uncharacterized protein YDR119W-A, named Cox26 (chain l in PDB 6HU9)
absent in _N. crassa_ genome

```
sp|Q2V2P9|YD19A_YEAST MFFSQVLRSSARAAPIKRYTGGRIGESWVITEGRRLIPEIFQWSAVLSVCLGWPGAVYFFSKARKA 66
```

Figure S5 Sequence alignment of *N. crassa* subunit Cox6a and *S. cerevisiae* subunit Cox13 with Clustal Omega (1.2.4), and sequence of *S. cerevisiae* subunit Cox26. Grey letters indicate residues that are absent in the *S. cerevisiae* model (PDB 6HU9), bold letters mark transmembrane regions. *S. cerevisiae* Cox6a has a homologue in *N. crassa* that is not present in the Cryo-EM map. *N. crassa* does not have a Cox26 homologue, and no density is visible in the Cryo-EM map.
